# Supplementary material for: Extracellular S100β Disrupts Bergman Glia Morphology and Synaptic Transmission in Cerebellar Purkinje Cells
Source: Brain Sci. 2019 Apr 12;9(4):80. doi: 10.3390/brainsci9040080 (PMC6523464; doi:10.3390/brainsci9040080)
Supplement: Supplementary file 1 [file brainsci-09-00080-s001.pdf]

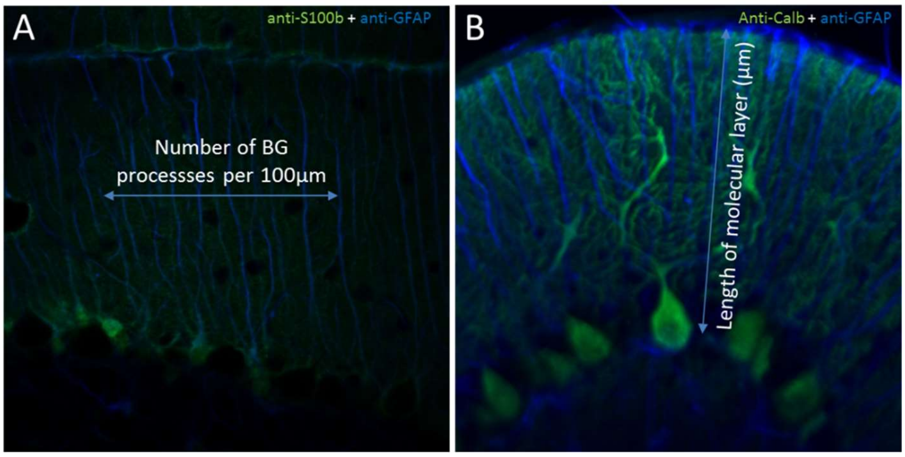

Figure S1. (**A**) Confocal image of S100β-injected area (anti-S100β and anti-GFAP staining). The line with two arrowheads illustrates the 100 μm length, where the parameters of BG (Bergmann glia) processes were evaluated. (**B**) Confocal image to illustrate staining with anti-Calbindin and anti-GFAP antibodies. The line with two arrowheads illustrates the thickness of the cerebellar molecular layer.

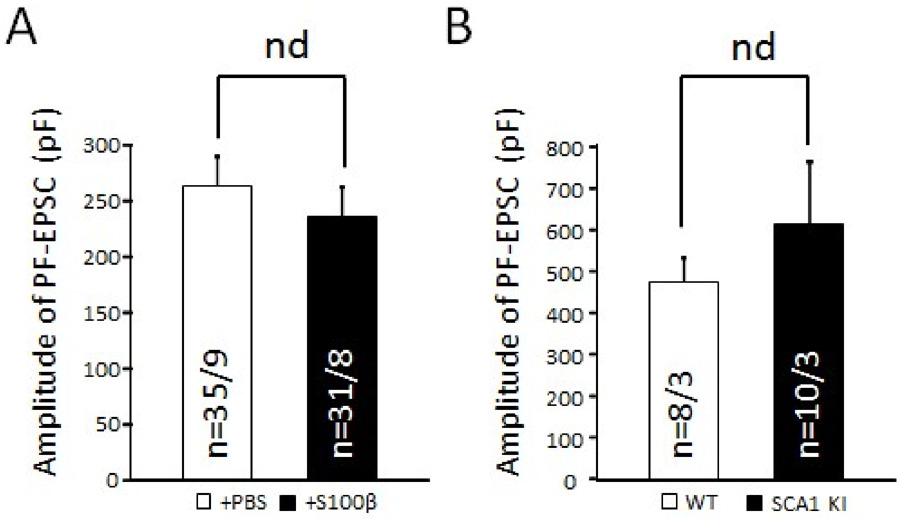

Figure S2. Electrophysiological characteristics of PCs. (**A**) The summary graph shows the average PF-EPSC amplitudes in PCs from PBS- and S100β-injected areas. There were no significant differences between the two groups. The numbers (n) of tested PCs and animals (PCs/animals) are indicated in the graph. (**B**) Average PF-EPSC amplitudes in PCs from three-week-old WT and SAC1 KI mice. Differences between the groups were not significant. The numbers (n) of tested PCs and animals (PCs/animals) are indicated in the graph.

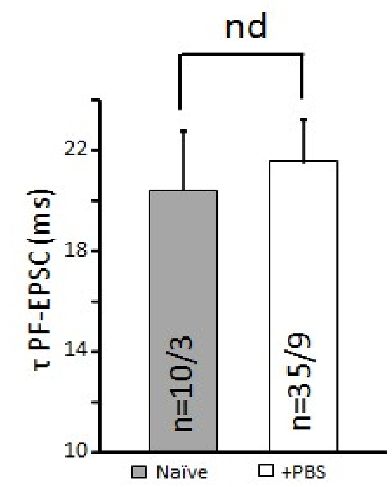

Figure S3. PBS injections do not alter decay time of PF-EPSC.
